# Putative β-Barrel Outer Membrane Proteins of the Bovine Digital Dermatitis-Associated Treponemes: Identification, Functional Characterization, and Immunogenicity

**DOI:** 10.1128/IAI.00050-20

**Published:** 2020-04-20

**Authors:** G. J. Staton, S. D. Carter, S. Ainsworth, J. Mullin, R. F. Smith, N. J. Evans

**Affiliations:** a Department of Infection Biology, Institute of Infection and Global Health, University of Liverpool, Leahurst Campus, Neston, Cheshire, United Kingdom; b Department of Livestock Health and Welfare, Institute of Veterinary Science, University of Liverpool, Leahurst Campus, Neston, Cheshire, United Kingdom; Washington State University

**Keywords:** *Treponema*, bovine digital dermatitis, outer membrane proteins, reverse vaccinology

## Abstract

Bovine digital dermatitis (BDD), an infectious disease of the bovine foot with a predominant treponemal etiology, is a leading cause of lameness in dairy and beef herds worldwide. BDD is poorly responsive to antimicrobial therapy and exhibits a relapsing clinical course; an effective vaccine is therefore urgently sought. Using a reverse vaccinology approach, the present study surveyed the genomes of the three BDD-associated *Treponema* phylogroups for putative β-barrel outer membrane proteins and considered their potential as vaccine candidates. Selection criteria included the presence of a signal peptidase I cleavage site, a predicted β-barrel fold, and cross-phylogroup homology. Four candidate genes were overexpressed in Escherichia coli BL21(DE3), refolded, and purified. Consistent with their classification as β-barrel OMPs, circular-dichroism spectroscopy revealed the adoption of a predominantly β-sheet secondary structure. These recombinant proteins, when screened for their ability to adhere to immobilized extracellular matrix (ECM) components, exhibited a diverse range of ligand specificities. All four proteins specifically and dose dependently adhered to bovine fibrinogen. One recombinant protein was identified as a candidate diagnostic antigen (disease specificity, 75%). Finally, when adjuvanted with aluminum hydroxide and administered to BDD-naive calves using a prime-boost vaccination protocol, these proteins were immunogenic, eliciting specific IgG antibodies. In summary, we present the description of four putative treponemal β-barrel OMPs that exhibit the characteristics of multispecific adhesins. The observed interactions with fibrinogen may be critical to host colonization and it is hypothesized that vaccination-induced antibody blockade of these interactions will impede treponemal virulence and thus be of therapeutic value.

## Abstract (precis)

Bovine digital dermatitis (BDD), an infectious disease of the bovine foot with a predominant treponemal etiology, is a leading cause of lameness in dairy and beef herds worldwide. BDD is poorly responsive to antimicrobial therapy and exhibits a relapsing clinical course; an effective vaccine is therefore urgently sought. Using a reverse vaccinology approach, the present study surveyed the genomes of the three BDD-associated *Treponema* phylogroups for putative β-barrel outer membrane proteins and considered their potential as vaccine candidates.

## INTRODUCTION

Bovine digital dermatitis (BDD) is a painful, ulcerative disease of the bovine foot and a significant cause of lameness in dairy cattle. Clinically, BDD presents as a malodorous, focally inflamed, circumscribed lesion of raised hyperkeratotic skin localized to the plantar/palmar aspect of the interdigital cleft, on or adjacent to the coronary band (1, 2). BDD is now considered the most common infectious cause of lameness in dairy cattle herds in the Northern Hemisphere and one of the most significant challenges to farm animal welfare. Moreover, the economic burden to the dairy industry resulting from production losses is considerable (3). In the United Kingdom, BDD is endemic, affecting an estimated 79% of dairy farms (4), and emergence of BDD in beef herds has also been recently described (5, 6). In addition, a new variant (contagious ovine digital dermatitis), noted for its particularly severe presentation in sheep, continues to spread through the United Kingdom’s national flock (7, 8). This disease therefore represents an additional and growing challenge to global food security.

A substantial body of evidence supports the involvement of multiple *Treponema* spp. at various stages of BDD lesion development (9–12). Three treponeme taxa in particular have been consistently isolated from lesion biopsy material from cases in the United States and the United Kingdom, namely, the Treponema medium phylogroup, the Treponema phagedenis phylogroup, and Treponema pedis (13, 14). The presence of these organisms deep within the lesion (15, 16), their clear association with necrotic changes in infected tissue (17), a failure to isolate these organisms from the feet of healthy animals (12, 18), and a disease-associated, specific IgG antibody response to these organisms (19–23) strongly imply an etiopathogenic role in BDD.

Treponemes are Gram-stain-negative bacteria exhibiting a spiral morphology and consist of an outer membrane (OM) that surrounds the axial filaments and the protoplasmic cylinder (24). The OM of these extracellular pathogens is a feature of considerable interest, given its surface exposure and the subsequent involvement of its components in host-pathogen interactions. Adhesins embedded in the OM play a critical role in bacterial cytoadherence to the host during colonization. The host extracellular matrix (ECM) is an important adherence target for pathogenic microorganisms during the primary stages of infection. Previous studies investigating the ECM binding capacity of the two most relevant human-pathogenic species, Treponema pallidum subsp. *pallidum*, the causative agent of syphilis, and Treponema denticola, a key member of the polymicrobial consortium implicated in periodontal disease, reported specific treponemal cell adherence to a range of immobilized ECM components, including fibronectin, laminin, fibrinogen, and collagen (25, 26). A growing number of adhesins are being identified and investigated to characterize the molecular basis of physical host-pathogen interactions (27–35). However, the fastidious nature of the BDD-associated treponemes has precluded any detailed characterization of the OM components likely to promote such interactions. Employing recently available BDD-associated treponeme genome sequences, we sought to identify novel β-barrel OM protein (OMP)-encoding genes and characterize the function and immunogenicity of the recombinantly expressed OMPs.

## RESULTS

### *In silico* detection of putative treponemal OMPs.

SignalP 4.1 analysis identified 182 *T. medium* T19 putative coding sequence (CDS) features predicted to contain an N-terminal peptidase I cleavage site. These features were further analyzed by three β-barrel prediction programs: BOMP, PRED-TMBB, and TMBETA-NET. CDS features predicted to encode β-barrel proteins by at least one these programs were selected for cross-phylogroup homology detection. In total, 15 CDS features identified in the *T. medium* T19 genome matched the following selection criteria precisely: (i) the presence of a signal peptidase I cleavage site, (ii) a predicted β-barrel topology, and (iii) cross-phylogroup homology (Table 1). Four CDS features, two from the *T. medium* genome and two from the *T. pedis* genome (including one homologous pair: C5N99_10335 and DYQ05_13425; amino acid sequence identity, 31.87%), were subsequently selected to evaluate the ability to bind to selected ECM components and to induce an IgG antibody response in calves.

**TABLE 1:** Bioinformatic analysis of four putative treponemal OMPs^*a*^

Putative OMP	*Treponema* phylogroup	Signal peptide cleavage site	β-Barrel and adhesin prediction				Homologous domain search		
			BOMP	PRED-TMBB	TMBETA-NET	SPAAN	PDB top hit (% probability; E value)	Function	Reference(s)
C5N99_02965	*T. medium*	Yes (20/21; LSA/QE)	Yes	No	Yes	Yes	OmpU (57.16; 120)	OM porin	87
C5N99_10335	*T. medium*	Yes (21/22; VFS/DG)	Yes	Yes	Yes	Yes	OmpA (97.71; 6.1e−5)	OM porin/adhesin	88, 89
DYQ05_13425	*T. pedis*	Yes (21/22; AFN/LS)	No	Yes	Yes	Yes	OmpA (98.07; 4.9e−6)	OM porin/adhesin	88, 89
DYQ05_06810	*T. pedis*	Yes (221/22; LSA/QT)	Yes	Yes	Yes	Yes	OprF (97.32; 0.0014)	OM porin	90

a Each putative treponemal OMP was selected on the basis of predicted β-barrel topology by at least one of the β-barrel prediction programs. The results generated by BOMP, PRED-TMBB, TMBETA-NET, and SPAAN were interpreted in accordance with default cutoff values. All four CDS features were predicted to share domain homology with known bacterial OMPs.

### Prediction of 3D tertiary structure.

**FIG 1:**
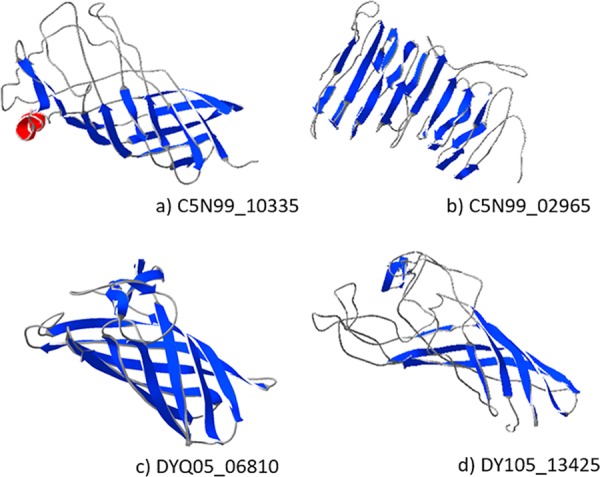
The four putative β barrel-outer membrane proteins (a to d) were structurally modeled using I-TASSER. A lateral view of the top-ranking ribbon model, as determined by the C score, is shown, with the β-sheet depicted in blue and α-helix depicted in red.

To generate predicted 3-dimensional (3D) structural models, each protein sequence was submitted to the I-TASSER server. The highest-ranking model for each protein is shown in Fig. 1. The four putative OMPs were each predicted to contain a typical β-barrel domain, consistent with localization to the outer membrane of Gram-negative bacteria.

Typical β-barrel structures were predicted for C5N99_10335, DYQ05_13425, and DYQ05_06810, whereas C5N99_02965 was predicted to adopt a β-solenoid fold (Table 2).

**TABLE 2:** I-TASSER structural modeling of the treponemal OMPs: a summary of results^*a*^

Putative OMP	C score^*b*^	Predicted topology	PDB structural analog (bacterial species)	Structural analog function	Reference
C5N99_10335	3.98	8-Stranded β-barrel	OmpT (E. coli)	Protease	91
C5N99_02965	2.73	β-Solenoid barrel	Serine-rich repeat protein (Lactobacillus reuteri)	Cell adhesion	92
DYQ05_13425	4.07	8-Stranded β-barrel	OprG (Pseudomonas aeruginosa)	Porin	93
DYQ05_06810	2.73	8-Stranded β-barrel	OmpA (E. coli)	Porin	94

a The amino acid sequence of each putative OMP was submitted to I-TASSER for 3D structural modeling and Protein Databank (PDB) structural analogue detection.

b C score: confidence score for estimating the quality of predicted models.

### Heat modifiability assay.

**FIG 2:**
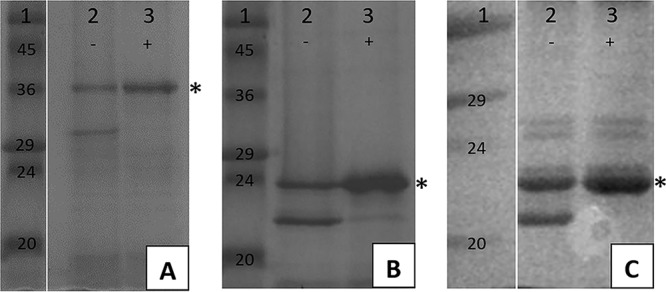
(A) C5N99_02965; (B) C5N99_10335; (C) DYQ05_06810. Lanes 1, wide-range MW marker (values are in kilodaltons); lanes 2, unheated sample; lanes 3, heated sample. The heat-modified (unfolded) forms of the proteins are distinguished from the unmodified (folded) forms by the addition of an asterisk. In panels A and C, the lane between marker and sample contained a wash fraction and has been removed for brevity.

Three of the proteins exhibited a heat-modifiable electrophoretic mobility, consistent with the stability of a protein comprised predominantly of a β-structure (36). Figure 2 shows the change in electrophoretic mobility observed under unheated versus heated conditions for C5N99_10335, C5N99_02965, and DYQ05_06810.

Heat modification of C5N99_02965 (Fig. 2A) led to a change in electrophoretic mobility and a change in the apparent molecular weight (MW), from ∼30 to ∼36 kDa. Similar changes in mobility were observed for C5N99_10335 (∼22 to ∼24 kDa [Fig. 2B]) and DYQ05_06810 (∼21 to 23 kDa [Fig. 2C]). Heat modifiability was not identified for DYQ05_13425 (data not shown).

### CD spectroscopic analysis of the treponemal recombinant OMP secondary structure.

**FIG 3:**
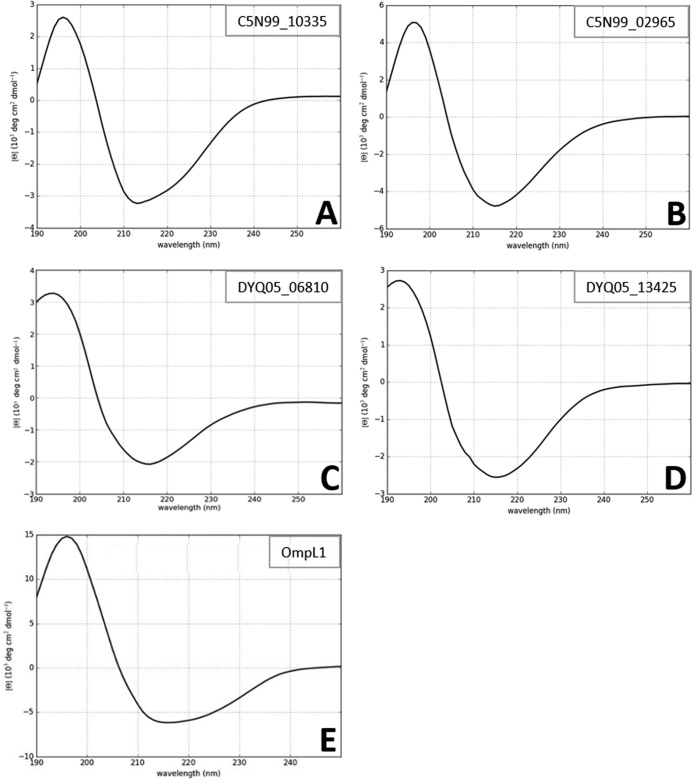
CD spectra of proteins encoded by C5N99_10335 (A), C5N99_02965 (B), DYQ05_06810 (C), DYQ05_13425 (D), and L. interrogans OmpL1 (E) are shown. Far-UV CD spectra are presented as an average of three scans recorded from 190 to 260 nm. φ, molar ellipticity.

Far-UV circular dichroism (CD) spectroscopy was employed to provide further evidence of the secondary structure fold of these proteins (Fig. 3). Consistent with a predicted β-barrel tertiary structure state, analysis of the four putative OMPs yielded spectral signatures typical of a predominantly β-sheet secondary structure, with spectral minima occurring at approximately 218 nm for C5N99_10335, C5N99_02965, DYQ05_06810, and DYQ05_13425 as well as the recombinant control protein, OmpL1.

### Serological response to putative OMPs during natural BDD infection.

**FIG 4:**
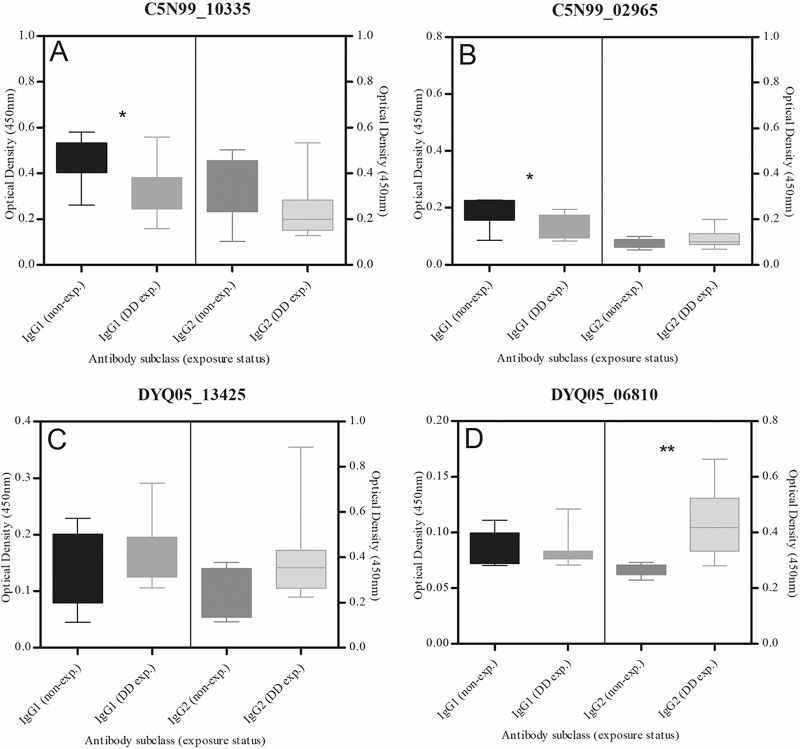
Serological assessment of the IgG1 and IgG2 responses to putative treponemal OMPs by ELISA. Error bars indicate standard errors of the means. Non-exp., nonexposed; DD exp., digital dermatitis exposed. Asterisks indicate a significant difference in IgG seroreactivity relative to control sera as determined by Mann-Whitney U test (*, *P* < 0.05; **, *P* < 0.005).

As demonstrated in Fig. 4, IgG2 seroreactivity to DYQ05_06810 was detected in BDD-infected Holstein Friesian cows (*n* = 12; 75%) relative to healthy control animals, with no apparent IgG2 response observed against the remaining three putative OMPs. No IgG1 response against any of the putative OMPs under investigation was identified. A statistically significant decrease in the IgG1 enzyme-linked immunosorbent assay (ELISA) optical densities (ODs) was observed in the sera of BDD-exposed animals, relative to controls, in the T. *medium* T19 putative OMP analyses (*P* < 0.05).

### Binding of the treponemal OMPs to ECM components.

**FIG 5:**
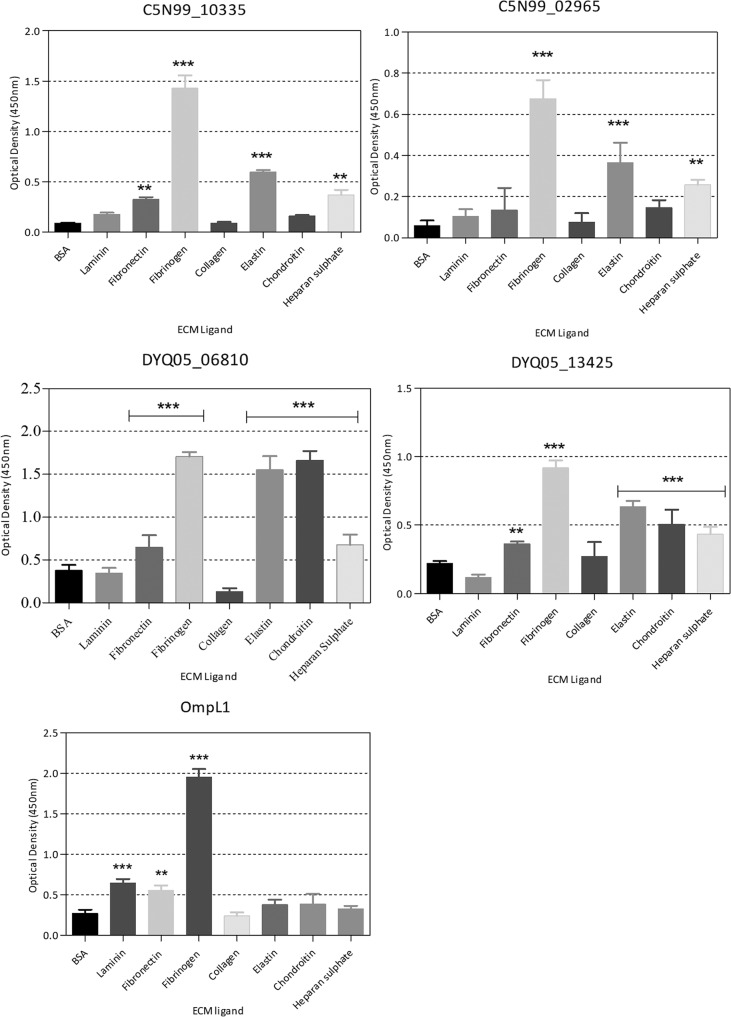
Immobilized ECM component binding screens of four putative β-barrel OMPs and the positive-control protein (OmpL1). Bars represent the mean OD of three independent experiments. Error bars indicate standard errors of the means. Asterisks indicate a significant difference in binding compared to the negative-control protein, BSA, as determined by one-way analysis of variance (ANOVA) and the Dunnett posttest (*, *P* < 0.05; **, *P* < 0.005; ***, *P* < 0.001).

Statistically significant (*P* < 0.05) adherence to fibrinogen, elastin, and heparan sulfate was observed for both C5N99_10335 and C5N99_02965, with C5N99_10335 additionally adhering to fibronectin (Fig. 5).

The *T. pedis* homologue of C5N99_10335, namely, DYQ05_13425, exhibited a binding profile similar to that of C5N99_10335 but was found to additionally bind to chondroitin. DYQ05_06810 bound to fibronectin, fibrinogen, elastin, chondroitin, and heparan sulfate.

**FIG 6:**
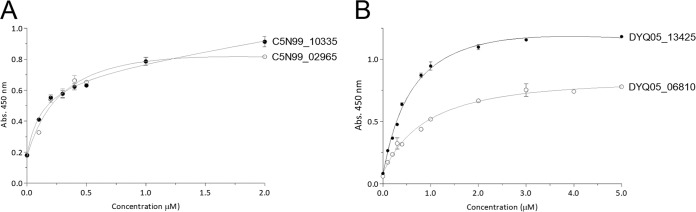
Binding affinity curves of the four putative β-barrel OMPs to bovine fibrinogen. (A) Putative OMPs from *T. medium*; (B) putative OMPs from *T. pedis*. Data points represent the mean ODs of replicate readings. Error bars indicate standard errors of the means.

Next, given the ubiquitous fibrinogen binding among these putative treponemal OMPs (*P* < 0.01), we sought to further characterize this interaction across a concentration range. The results of these analyses are shown in Fig. 6.

The binding interactions between fibrinogen and C5N99_10335, C5N99_02965, DYQ05_06810, and DYQ05_13425 were observed to be concentration dependent. Binding saturation levels were achieved with recombinant protein concentrations of ∼1 μM, ∼1.5 μM, and ∼2.0 μM for C5N99_02965, DYQ05_13425, and DYQ05_06810, respectively. Conversely, C5N99_10335, although showing a tendency toward reaching saturation, failed to do so up to a concentration of 2 μM. Further examination of this interaction was precluded by insufficient protein yield. Dissociation constant (*K_d_*) values were estimated by nonlinear regression analysis of the binding curves. Table 3 summarizes the *K_d_* values calculated from these experiments. The positive-control protein, OmpL1, was similarly observed to adhere to bovine fibrinogen in a dose-dependent and saturable manner, as previously reported (37) (data not shown).

**TABLE 3:** Binding interactions between the putative recombinant OMPs and bovine fibrinogen

OMP	Dissociation constant
C5N99_02965	0.3370 ± 0.09753
DYQ05_13425	0.7180 ± 0.08743
DYQ05_16810	1.024 ± 0.2946
OmpL1	0.3669 ± 0.04328 (0.223 ± 0.063) (47))

### Far Western blotting.

**FIG 7:**
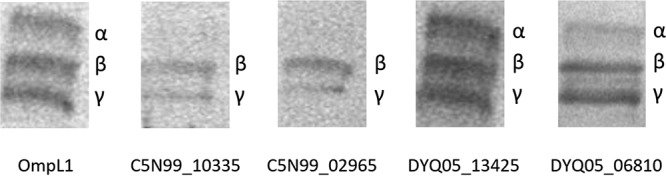
Specific binding interactions between the recombinant proteins and the α, β, and γ chains of bovine fibrinogen, using monoclonal anti-polyhistidine antibody as the probe.

Far Western blotting identified interactions between the putative OMPs and individual chains of the fibrinogen molecule. The results of this analysis are shown in Fig. 7.

The results of the far Western analysis revealed that DYQ05_13425 and DYQ05_06810 interacted with all three chains of the fibrinogen molecule, similarly to OmpL1. Conversely, C5N99_10335 and C5N99_02965 were observed to interact with the β and γ chains of fibrinogen only.

### Immunogenicity.

The immunogenic properties of these four fibrinogen-binding proteins were investigated following inoculation into two naive bull calves as part of a multivalent antigen cocktail. ELISA analysis of prevaccination sera yielded broadly comparable ELISA ODs between calves.

**FIG 8:**
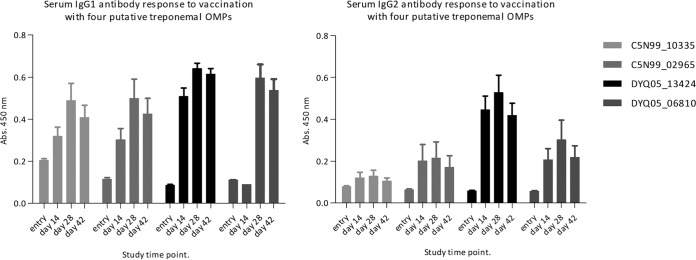
The serological IgG1 and IgG2 responses to four treponemal putative OMPs were assessed in BDD-naive calves (*n* = 2). The mean ODs of both immunized calves are shown. Error bars indicate standard errors of the means.

This pilot immunogenicity trial demonstrated that this subcutaneous prime-boost vaccination protocol, involving an aluminum hydroxide-adjuvanted 100-μg dose of each treponemal recombinant OMP, was capable of eliciting IgG antibodies in BDD-naive bull calves (Fig. 8). All animals vaccinated with the recombinant protein cocktail seroconverted rapidly, permitting treponemal OMP-specific IgG antibody detection by day 14. A second booster vaccination on day 14 enhanced the IgG response further, and specific antibody titers peaked at day 28. No seroconversion was detected in the control animals up to day 56 (the last day of the trial; data not shown). The fold change, calculated as the mean OD change on day 28 from preimmunization baseline (Table 4), was used to account for varying baseline ELISA ODs.

**TABLE 4:** Fold change in calf seroreactivity to the inoculated treponemal recombinant putative OMPs

Antigen	IgG1 fold change (day 28 postimmunization)	IgG2 fold change (day 28 postimmunization)
C5N99_10335	2.37	1.66
C5N99_02965	4.34	3.58
DYQ05_13425	7.44	9.10
DYQ05_06810	5.42	6.00

While all recombinant proteins were shown to be immunogenic under the conditions of this trial, C5N99_10335 was found to be the weakest driver of both IgG1 and IgG2 antibody responses, with ELISA OD fold changes calculated to be 2.37 and 1.66 from baseline, respectively. DYQ05_13425, conversely, was found to be the most potent immunogen, with ELISA OD fold changes from baseline calculated to be 7.44 and 9.10, for IgG1 and IgG2, respectively. The specificity of the antisera was confirmed using Western blotting and revealed the presence of treponemal putative OMP-specific IgG1 and IgG2 serum antibodies in immunized animals only (data not shown). Serological analysis on day 42 of the trial revealed a slight waning of the IgG antibody response in all cases. The trial vaccine was well tolerated in vaccinated calves.

## DISCUSSION

Using a bioinformatics-based approach, the present study sought to identify and characterize novel BDD-associated treponemal OMPs, bearing in mind the potential value of these molecules as BDD vaccine candidates. Postpurification analysis of the four refolded proteins by CD spectroscopy yielded spectra indicative of a predominantly β-strand secondary structure with three of the proteins (C5N99_10335, C5N99_02965, and DYQ05_06810) additionally being demonstrated to be heat modifiable. These findings are consistent with the characteristics of proteins with a β-barrel tertiary structure. Conversely, DYQ05_13425 was found to not be heat modifiable. Owing to a highly resistant tertiary structure, some bacterial OMPs exhibit an unusual stability to heat in the presence of SDS and an extended heating period may be required to convert such proteins to their denatured form, as reported for protein F from Pseudomonas aeruginosa (38) and OmpL1 from Leptospira interrogans (36).

Examination by ELISA of IgG seroreactivity in animals naturally infected with BDD revealed that only one protein (DYQ05_06810) was capable of discriminating clearly between the sera of animals with recent or active BDD infection and cattle presumed not to have been exposed to the BDD treponemes. In identifying a disease-specific IgG2 antibody response to DYQ05_06810, these findings are in concordance with the findings of a number of previous studies that demonstrated a strong antitreponemal IgG2 bias in the antibody response of animals naturally infected with BDD (23, 39, 40). These data suggest that while DYQ05_06810 is accessible to the immune system during treponemal infection (and thus capable of eliciting IgG2 antibodies), the remaining three putative treponemal OMPs may be expressed as subdominant antigens or their expression may be immunosubversive in nature. Unexpectedly, a reduction in OD was observed when comparing the IgG1 responses to the two *T. medium* T19 putative OMPs in cows exposed to BDD, relative to healthy controls. The reason for this is unclear, although treponemes exhibit a number of immunosuppressive functions capable of interfering with both innate (41) and adaptive (42) immune activities, the utilization of which likely contribute to infection chronicity. In this case, it is speculated that whereas previous colonization with bacteria expressing orthologues of C5N99_10335 and C5N99_02965 leads to the production of cross-reactive antibodies (which are detectable in control animals), subsequent infection with *T. medium* leads to a reduction in the serotiters of these antibodies via immunosuppressive mechanisms. Crucially, both C5N99_10335 and C5N99_02965 have been detected in the transcriptome of BDD lesions (43), and studies are required to investigate their capacity to modulate host immunity.

Presently, no serological assay to detect BDD treponemes is commercially available for field diagnostics, and immunoassay-based serological assessment of BDD in research settings is currently dependent on the use of whole-cell lysates. The *T. phagedenis* putative proline-rich repeat lipoprotein PrrA was previously identified as an immunogenic protein of *T. phagedenis*-like strain V1 (isolated from a BDD lesion) and as an antigenic target capable of discriminating between animals acutely infected with BDD (*n* = 8) and BDD-naive animals (*n* = 7) (44). An ELISA capable of detecting bovine anti-PrrA antibodies in serum and milk has been available to the research community for some time, although there are currently no reports detailing postmarketing experience. In addition, the PrrA gene and its product are from several *T. phagedenis*-like BDD lesion isolates (44, 45), potentially limiting the value of this ELISA as a means of determining herd infection status. It is therefore proposed that the IgG2 antibody response to DYQ05_06810 be further evaluated as a potential antigenic marker of seroconversion in BDD-infected animals. The diagnosis of BDD currently remains restricted to clinical examination of the lifted foot by means of individual restraint (46), and specificity and sensitivity are limited by subjectivity and observer bias (47). A quantitative diagnostic ELISA is less labor-intensive, is less time-consuming, and is a more reproducible indicator of biological infection (48). Since these recombinant molecules can be readily synthesized in E. coli expression systems, the difficulties in cultivating BDD-associated *Treponema* spp. for whole-cell antigen preparations are bypassed.

An initial ECM binding screen demonstrated that the putative treponemal β-barrel OMPs identified in this study exhibited ECM-adhesive properties, supporting their role in bacterial cytoadherence to host tissues. Interestingly, these proteins exhibited multispecific ECM binding profiles. One of the most striking properties of the putative treponemal OMPs was an ability to adhere to immobilized bovine fibrinogen. Because of the highly statistically significant nature of this interaction, these fibrinogen binding activities were investigated further and found to be concentration dependent and saturable, suggesting the existence of fibrinogen-specific binding sites. The estimated dissociation constants (*K_d_*s) (0.34 and 0.72 for C5N99_02965 and DYQ05_13425, respectively) broadly align with those calculated for other spirochetal fibrinogen binding proteins, including the leptospiral proteins OmpL1 (0.223 μM) (37), OmpL37 (0.244 μM) (49), and Lsa33 (0.12 μM) (50), supporting the relevance of these interactions *in vivo*. DYQ05_06810 exhibited a higher estimated *K_d_*, 1.0 μM, which is of a magnitude similar to those of other biologically relevant ECM binding interactions, including those reported for OmpL1 interactions with laminin (*K_d_* = 2.10 μM) and fibronectin (*K_d_* = 1.24 μM) (51).

Bovine fibrinogen is a 340-kDa dimeric glycoprotein comprising of three pairs of nonidentical Aα (∼67 kDa), Bβ (∼55 kDa), and γ (∼48 kDa) peptide chains. Fibrinogen is a major clotting factor and performs an essential role in preventing hemorrhage and facilitating vascular repair. At sites of tissue damage, fibrinogen is found embedded with the extracellular matrix (52). It has previously been demonstrated that successful and consistent experimental transmission of BDD requires abrasion of the skin (53). Abrasive trauma would be expected to lead to enhanced fibrinogen deposition, and it is therefore hypothesized that ECM-associated fibrinogen represents an adherence target during the initial stage of host colonization. In support of this hypothesis, it has been demonstrated previously that two *T. medium*-like treponemal strains, UB1467 and UB1090, isolated from a bovine and an ovine DD lesion, and a *T. pedis*-like strain, UB1466, isolated from an ovine DD lesion, were all capable of adhering to immobilized fibrinogen (54). Thus, adhesin-mediated treponemal adherence to the host via such interactions may be central to the treponemal infection, in which abrasive trauma may be necessary point of entry.

Soluble host proteins, including plasma fibrinogen, may act as a diffuse peptide nutrient source within inflamed tissues. However, fibrinogen additionally plays a direct role in antimicrobial host defense. First, thrombin-catalyzed conversion to insoluble fibrin clots leads to the formation of a structural protective barrier capable of containing bacteria and preventing dissemination (55). Second, this conversion to thrombin leads to the release of potent chemotactic elements, including fibrinopeptide B, which drives an influx of phagocytes (56). Among the treponemal OMPs, chymotrypsin-like protease (CTLP) from T. denticola and Tp0751 (Pallilysin) from T. pallidum subsp. *pallidum*, are both capable of adhering to fibrinogen while additionally exhibiting fibrinogenolytic protease activity, with important deleterious consequences for platelet homeostasis and clot formation (29, 57). BDD lesions tend to bleed readily upon palpation (58), and a disturbance in the coagulation pathway arising from treponemal fibrinogen targeting is suspected. The interactions observed between the recombinant proteins and specific chains of the fibrinogen molecule identified in the present study may support this hypothesis, since the four recombinant proteins exhibited the capacity to adhere to all three fibrinogen chains or the Bβ and γ chains only. Crucially, platelet aggregation is dependent on the binding of platelet membrane glycoprotein IIa/IIIb to the fibrinogen Aα and γ chains (59), and previous studies have identified selective fibrinogen chain targeting by pathogenic bacterial OMPs. For instance, targeting of the γ chain of fibrinogen has been previously reported for Staphylococcus aureus clumping factor A (60); this targeting leads to disturbances in platelet aggregation, fibrin clot formation, and platelet-mediated clot retraction (61).

The mechanisms that underpin the observed interactions with fibrinogen are unknown and require further investigation. Further studies, using gene mutagenesis and critical binding domain mapping, are required to fully understand the molecular basis for these interactions. It is noteworthy that these proteins appear to adhere to a number of other ECM components, demonstrating the apparent multispecificity of these putative treponemal adhesins. Although we have not yet explored these interactions further, this suggests that these molecules may be functionally similar to a number of other spirochaetal OMPs capable of adhering to multiple ligands, including Tp0751 (29) and OmpL1 (51). This presumably represents an evolutionary adaptation that minimizes bacterial surface immunogenicity while preserving adhesive function.

Given the apparent importance of these putative OMPs to fibrinogen (and other ligands), and their potential roles in host colonization and pathogenicity, we sought to assess their immunogenic properties. Previous studies have shown that anti-adhesin antibodies, elicited by vaccination, have the potential to protect the host (62, 63), and it is hypothesized that blockade of these putative OMPs would impede host colonization and/or virulence. Previous attempts at designing a BDD vaccine have been limited to the use of treponemal whole-cell lysate “bacterin” formulations of a single phylotype, and field trials have been disappointing (64). The subcutaneous vaccine formulation evaluated in the present study was designed specifically to induce an IgG response against putative treponemal fibrinogen-binding OMPs, which may be critical to host colonization yet exist as subdominant antigens with little or no immunogenic capacity in natural infection.

Since recombinant proteins tend to be relatively weak antigens (65), aluminum hydroxide was employed as an adjuvant. Both IgG1 and IgG2 responses to the putative OMP antigens were generated for the four proteins tested. Moreover, fold change in the ELISA ODs generated for IgG1, relative to IgG2, was generally greater. Aluminum compounds are considered to be principally promoters of Th2 polarization, at least in humans and mice (66, 67), and may explain the IgG1 subclass bias observed in this study. A mixed IgG1/IgG2 or IgG1-polarized host response may prove to be an important correlate of protection against an infection that is usually considered to induce a nonprotective, yet robust, IgG2 response (23, 39, 40). However, because a later study identified IgG1 as the predominant IgG subclass in cattle both naturally exposed to BDD and experimentally infected with BDD-associated treponemes (20), there exists considerable uncertainty of the nature of the bovine immune response in BDD and, consequently, its potential for manipulation.

When adjuvanted with aluminium hydroxide, the four proteins under investigation were found to be immunogenic, although C5N99_10335 was found to be a comparatively weak immunogen, highlighting the heterogeneity of the immune response. Whether this apparent variation in immunogenicity has arisen from intrinsic differences in the molecular structures of these recombinant proteins, host-specific variations in immune response, or potential contamination with endogenous endotoxin has yet to be established. Moreover, while the immunogenic potential of these putative OMPs has been demonstrated in this study, neither the duration of the IgG response nor its magnitude (in terms of absolute antibody titer) was quantified. Since the immunized calves were not assessed for postvaccination susceptibility to BDD, it is unknown whether high titers of the IgG antibodies generated during this study correlate with protection against disease, and future studies are warranted. However, these data indicate successful B cell priming after the initial vaccination and a boost effect following the second vaccination, both of which are important characteristics of a vaccine component.

Given the difficulties associated with the isolation, cultivation, and purification of the BDD treponemes, the development of a vaccine against BDD has previously been substantially hindered. This *in silico* approach to novel OMP identification overcomes the challenges of traditional vaccine design methods. To this end, we report on the identification and characterization of four putative adhesins, selected from the sequenced genomes of *T. medium* and *T. pedis* phylogroups, two of the principal treponeme taxa associated with BDD. Further studies are justified to establish their value as BDD vaccine components.

## MATERIALS AND METHODS

### Ethical approval.

All experimental work involving animals was covered by UK Home Office Project License PPL 70/8330.

### *In silico* identification of OMPs.

Previously generated and annotated representative genomes of the three BDD treponemes, *T. medium* T19 (accession number CP027017), *T. phagedenis* T320A (accession number CP027018) and *T. pedis* T3552B^T^ (accession number CP045670) were subjected to *in silico* analysis to identify putative OMPs via prediction of encoded β-barrel structural motifs. Putative coding sequence (CDS) features for each genome were translated to their amino acid sequences using Artemis (68). All translated *T. medium* T19 CDS features were analyzed for the presence of a signal peptidase I cleavage site using SignalP 4.1 (69). Sequences predicted to harbor a signal peptide were further scrutinized for signatures of β-barrel tertiary structure using three β-barrel prediction programs (BOMP [70], TMBETA-NET [71], and PRED-TMBB [72]). All *T. medium* T19 CDS features which were predicted to code a β-barrel tertiary-structured protein by at least one of the β-barrel prediction programs were retained. Homologues of putative *T. medium* T19 OMPs were identified in *T. phagedenis* T320A and *T. pedis* T3552B^T^ genomes using a combination of a Markov cluster algorithm (73) and BLAST (74) and their OMP predictions verified independently. Putative OMP sequences which were conserved in all three genomes were examined for predicted adhesin functionality using SPAAN (75) and their tertiary structures modeled using I-TASSER (76).

### Cloning and expression of candidate antigens.

*T. medium* T19, *T. phagedenis* T320A, and *T. pedis* T3552B^T^, isolated previously from BDD lesion biopsy specimens (12, 14) and cryopreserved in 10% (vol/vol) glycerol at –80°C, were cultured as described previously (12). Genomic DNA (gDNA) was extracted from the treponeme cultures at late exponential phase using Chelex 100 resin (Bio-Rad Laboratories Ltd., Hemel Hempstead, UK) according to a previously described method (77). The Gateway system (Life Technologies, Paisley, UK) was utilized for gene cloning and expression. Putative OMP sequences, lacking their signal peptide sequences, were amplified from the gDNA using high-fidelity Phusion polymerase (Thermo Scientific, Hemel Hempstead, UK) in accordance with manufacturer instructions. Primers (Table 5) for amplification contained CACC overhangs to facilitate entry cloning. A well-characterized OMP (OmpL1) from Leptospira interrogans serovar Copenhageni strain M20 was selected (37, 51, 78) and produced as a recombinant protein control.

**TABLE 5:** Primers used to amply putative OMP genes for recombinant expression

Putative OMP locus tag	*Treponema* phylogroup	Primer sequence (5′–3′)	Predicted band size (kb)	Predicted mass (kDa)
C5N99_10335	*T. medium*	Forward: CACCGATGGGGTCGATTTTTCG	0.7	27.1
		Reverse: CTACAGCTTAAAAGCGATCC		
C5N99_02965	*T. medium*	Forward: CACCCAGGAAGAAGGAGCAGAGG	0.9	35.0
		Reverse: AGAGATACCCATTAGTTGTTG		
DYQ05_13425	*T. pedis*	Forward: CACCTTAAGCGATATTTCAGGCGATG	0.8	29.8
		Reverse: TTACAGCTTCCATGCAATACC		
DYQ05_06810	*T. pedis*	Forward: CACCGCAAAGACTATCGGTCTTAATTTG	0.9	22.1
		Reverse: TTAAAAATAAACTCTTAAACCCGC		
OmpL1	L. interrogans	Forward: CACCAAAACATATGCAATTGTAGGATTTG	0.9	31.0
		Reverse: TTAGAGTTCGTGTTTATAACCG		

Amplified putative OMPs were inserted into the Gateway entry plasmid pENTR/d-TOPO (Life Technologies, Paisley, UK) in accordance with the manufacturer’s instructions prior to chemical transformation into Escherichia coli Top10 cells. Positive transformants were selected on LB agar plates containing kanamycin (50 μg/ml) and plasmid DNA from successful transformants isolated using the Qiagen plasmid miniprep kit (Qiagen, Manchester, UK). Successful amplicon insertion was confirmed using EcoRV restriction digest analysis (Thermo Fisher, Horsham, UK). Inserts were thereafter cloned into the Gateway expression vector pDEST17, using a site-directed integration reaction in accordance with the manufacturer’s instructions (Life Technologies, Paisley, UK) prior to chemical transformation into E. coli DH5α. Positive transformants were selected on LB agar plates containing ampicillin (100 μg/ml) and the plasmid DNA isolated as previously described. pDEST17-gene constructs verified by EcoRI endonuclease restriction digest analysis and Sanger sequencing (Source Bioscience, Nottingham, UK).

### Protein expression, refolding, and purification.

All protein expression was performed in E. coli BL21(DE3) (Life Technologies, Paisley, UK). E. coli BL21(DE3) cultures were grown at 37°C with shaking in LB medium (2 liters) containing ampicillin (100 μg/ml), until the OD at 600 nm (OD_600_) was 0.8 to 1. Protein expression was induced by the addition of 1 mM isopropyl-β-d-thiogalactopyranoside (IPTG; Sigma-Aldrich, Gillingham, UK). Cultures were grown for a further 4 to 5 h and cells harvested by centrifugation (3,500 × *g*, 4°C, and 10 min). E. coli BL21(DE3) cell pellets were resuspended in 50 mM Tris-HCl (pH 7.9) (20 ml per 10 g [wet weight] of cells) containing lysozyme (5 mg/g [wet weight] of cells; Sigma-Aldrich, Gillingham, UK) and incubated on ice for 30 min prior to cellular disruption using a microsonicator tip (Soniprep-150, MSE, London, UK). Inclusion bodies (IB) containing recombinant proteins were subsequently harvested by centrifugation (10,000 × *g*, 4°C, and 30 min). IB pellets were resuspended in 150 ml of IB detergent buffer (4% [vol/vol] Tergitol 15-S-9 [Sigma-Aldrich, Dorset, UK], 50 mM Tris HCl [pH 7.9]) with rapid stirring for a minimum of 2 h, washed twice in 150 ml of IB wash buffer (50 mM Tris HCl [pH 7.9]), and resuspended in solubilization buffer (6 M guanidine hydrochloride, 50 mM Tris-HCl [pH 7.9], and 1 mM EDTA; 40 ml per 500 mg of IB) for 1 h with constant agitation. The suspension was centrifuged (10,000 × *g*, 4°C, and 30 min) to remove insoluble material. Recombinant protein refolding was performed by rapid dilution (79) into a refolding buffer (250 mM NaCl, 50 mM Tris-HCl [pH 7.9], 5% *N*,*N*-dimethyldodecylamine *N*-oxide solution [LDAO; Sigma-Aldrich, Dorset, UK]) which was subsequently dialyzed against 10 volumes of dialysis buffer (250 mM NaCl, 50 mM Tris-HCl [pH 7.9], 0.1% LDAO). Refolded, recombinant proteins were purified by standard immobilized metal affinity chromatography (80), sterilized through a 0.2-μm filter, and stored at –80°C. The purity of the recombinant proteins was assessed by SDS-PAGE.

### Evaluation of secondary structure.

**(i) Heat modifiability.** The sensitivity of the recombinant proteins to denaturation upon heating was determined by comparing the electrophoretic mobilities of the refolded recombinant proteins prepared in SDS sample buffer without reducing agent (100 mM Tris-HCl [pH 6.8], 4% [wt/vol] SDS, 0.2% [wt/vol] bromophenol blue, 20% [vol/vol] glycerol) and either incubated at ambient temperature for 10 min or heated to 100°C for 10 min prior to SDS-PAGE analysis, as described previously (81).

**(ii) CD spectroscopy.** Far-UV circular-dichroism (CD) spectroscopy was performed using a Jasco J-810 spectropolarimeter (Japan Spectrocopic, Tokyo, Japan) equipped with a Peltier unit for temperature control. Spectra were measured from 190 to 260 nm using a 1-mm path length cell at intervals of 0.5 nm and presented as an average of three scans. Spectra were analyzed by Beta Structure Selection (BestSel) software (http://bestsel.elte.hu/) (82) to calculate the percent secondary-structure content from the ellipticity experimental data.

### Evaluation of immunogenicity during natural infection.

**(i) Cattle sera.** An ELISA was performed to investigate systemic IgG seroreactivity to the putative OMPs in blood samples collected from cows naturally infected with BDD. Sera from 16 adult Holstein-Friesian cows with a recent (<6-month) history of BBD were collected from a dairy herd situated in Cheshire, UK. Similarly, sera from 5 healthy adult Holstein-Friesian cows were collected from a closed dairy herd situated in Monmouthshire, UK, and were included as a control group. In all cases, whole blood was collected from the coccygeal vein. Following clotting and centrifugation, the serum fraction was harvested and stored at –20°C for serological assessment.

**(ii) Serological ELISA.** Nonactivated, 96-well microtiter plates (Microplate Immulon 2HB; Thermo Scientific, Hemel Hempstead, UK) were coated with a single recombinant protein (5 μg/ml) in phosphate-buffered saline (PBS; pH 7.2) and incubated for 1 h (37°C) and overnight (4°C). Unbound antigen was removed by washing with PBST (PBS-Tween 20; 0.05%). All sera were diluted 1/100 in PBST, pipetted into ELISA plate wells in duplicate, and incubated for 1 h (37°C). Wells were washed as before and incubated for 1 h (37°C) with 100 μl of monoclonal mouse anti-bovine immunoglobulin class G subclass 1 (IgG1) antibody, clone IL-A60 (Bio-Rad, Hemel Hempstead, UK), or monoclonal mouse anti-bovine immunoglobulin class G subclass 2 (IgG2) antibody, clone IL-A2 (Bio-Rad), diluted 1:1000. To ensure adherence of the antigen to the plate, 100 μl of mouse monoclonal anti-polyhistidine antibody, clone HIS-1 (Sigma-Aldrich, Dorset, UK), diluted 1:2,000 in PBST, was added to recombinant control wells. Following a washing, wells were incubated with 100 μl of horseradish peroxidase (HRP)-conjugated goat anti-mouse IgG antibodies (Sigma-Aldrich), diluted 1:10,000 in PBST for 1 h (37°C). Following washing, the presence of HRP-conjugated goat anti-mouse IgG antibodies was detected by the addition of 100 μl of the HRP substrate 3,3′,5,5′-tetramethylbenzidine (TMB; Sigma-Aldrich). The reaction was terminated after ∼20 min by the addition of 100 μl of 0.5 M hydrochloric acid. The optical density (OD) of each well was read at 450 nm using a microplate reader (Multiskan EX; Thermo Fisher Scientific, Loughborough, UK). All data were processed and analyzed using GraphPad Prism 5 (GraphPad, San Diego, CA). In order to classify results as positive or negative, an ELISA OD value of less than or equal to the mean plus 3 standard deviations of the control sera was considered to be nonreactive (83).

### Evaluation of adhesin function.

**(i) ECM macromolecules.** All ECM macromolecules were purchased from Sigma-Aldrich (Dorset, UK) and prepared from the following sources: collagen I from bovine skin, elastin from bovine neck filament, fibrinogen from bovine plasma, heparan sulfate from bovine kidney, chondroitin sulfate from bovine cartilage, and laminin-1 from the basement membrane of Engelbreth-Holm-Swarm mouse sarcoma.

**(ii) ECM binding ELISA.** An ELISA was performed to screen the recombinant proteins for the ability to attach to individual ECM macromolecules using a previously described method (31). Briefly, Immulon 2HB plates (Thermo Fisher, Horsham, UK) were coated with 5 μg/ml of the ECM component or the negative-control protein (bovine serum albumin [BSA]) by incubation for 1 h at 37°C and overnight at 4°C, washed with PBS containing 0.05% Tween 20 (PBST), and blocked with a 1% (wt/vol) BSA solution. Recombinant proteins, diluted in PBST, were added at 10 μg/ml to screen for ECM binding activity, and a range of concentrations (from 0 to 6 μM) was used to determine the dose dependency of these binding interactions. Following incubation, bound recombinant proteins were detected by addition of mouse monoclonal anti-polyhistidine IgG antibody (Sigma-Aldrich, Dorset, UK), diluted 1:2,000, before proceeding as described before. *K_d_* values were estimated from curves fitted by nonlinear regression analysis in GraphPad Prism v. 5, using the following equation: *K_d_* = (*A*_max_ [protein])/*A*) – [protein], where *A* is the absorbance at a given protein concentration, *A*_max_ is the maximum plate reader absorbance (when the equilibrium is reached), [protein] is the protein concentration, and *K_d_* is the dissociation equilibrium constant (84, 85).

### Far-Western blotting.

A far-Western blotting technique was employed to further characterize the specific binding interactions between the recombinant proteins and bovine fibrinogen (86). To dissociate native bovine fibrinogen into its constituent polypeptide chains (Aα, Bβ, and γ), 60 μl of bovine fibrinogen stock solution (1 mg/ml) was mixed with 350 μl of gel loading buffer (100 mM Tris-Cl [pH 6.8], 4% SDS, 0.2% bromophenol blue, 20% glycerol, 200 mM dithiothreitol), heated at 95°C for 5 min, and separated in Tris-glycine polyacrylamide gels by SDS-PAGE (4 to 20% gradient gel) at a constant voltage of 180 V for 50 min. The fibrinogen chains were electroblotted onto a nitrocellulose membrane (100 V, 240 mA, and 120 min), and the membrane was blocked with 5% (wt/vol) skimmed milk. Membranes were subsequently incubated with 30 μg/ml of recombinant protein. Any bound protein was detected by incubation of the membrane with mouse anti-polyhistidine antibody (Sigma-Aldrich, Dorset, UK), diluted 1:2,000, followed by goat anti-mouse antibody (Sigma-Aldrich) and development in 3,3′-diaminobenzidine membrane substrate (Sigma-Aldrich).

### Immunogenicity trial in calves.

**(i) Calves.** The immunogenicity of the recombinant proteins was evaluated in two Holstein-Friesian calves. A control group of two additional calves was used to verify that any serological response did not result from environmental exposure to BDD-associated treponemes or to ubiquitous antigens.

The four calves were reared and maintained according to routine agricultural practice at the university’s farm, with housing conditions having increased biosecurity to reduce the risk of exposure to the BDD-associated *Treponema* spp. Calves were bedded on straw and quarantined for 4 weeks before vaccine administration. Calves were bled immediately prior to vaccine administration to ascertain preimmunization antibody status.

**(ii) Formulation of the vaccine.** The multivalent vaccine was formulated to deliver the four recombinant proteins simultaneously and comprised of 100 μg of each recombinant protein and 40 μl of aluminum hydroxide adjuvant (Rehydragel, Chemtrade Logistics, Toronto, Ontario), adjusted to a final volume of 2 ml using PBS. All vaccines were administered subcutaneously to the left flank. Calves received an initial 2-ml dose of the vaccine followed by a 2-ml booster dose 2 weeks later. Concurrently, control animals (*n* = 2) received a 2-ml dose of the vehicle only. Blood samples, obtained by jugular venepuncture, were collected at 2-week intervals for 4 weeks beginning with a preimmunization draw at day zero. Serum was retained for serological studies.

**(iii) Detection of serum IgG antibodies by ELISA.** Vaccinee IgG1 and IgG2 antibody seroreactivity to the recombinant proteins was determined as described above. All data were processed and analyzed using GraphPad Prism 5 (GraphPad, San Diego, CA). ELISA reactivity was confirmed by Western blotting using previously described methods (23).
